# Difficult Cases of Odontogenic Deep Neck Infections: A Report of Three Patients

**DOI:** 10.4274/balkanmedj.2015.1379

**Published:** 2017-03-28

**Authors:** Onur İsmi, Mesut Yeşilova, Cengiz Özcan, Yusuf Vayisoğlu, Kemal Görür

**Affiliations:** 1 Department of Otorhinolaryngology, Mersin University School of Medicine, Mersin, Turkey

**Keywords:** Odontogenic infection, deep neck infection, abscess, tracheotomy, mediastinitis, complication

## Abstract

**Background:**

Deep neck infections are important otolaryngologic emergencies due to serious complications and the risk of airway compromise, which can lead to mortality. Although the most common causes among pediatric patients are tonsillitis and pharyngeal infections, odontogenic infections are an important cause in adults.

**Case Report:**

We present three patients with multiple deep neck space abscess formation due to odontogenic infection. Two of them required tracheotomy due to airway compromise, and one had mediastinitis.

**Conclusion:**

An underestimated tooth infection can cause hazardous complications such as mediastinitis and respiratory distress requiring tracheotomy.

Deep neck infections (DNIs) are important otolaryngologic emergencies requiring prompt diagnosis and treatment to prevent hazardous complications such as mediastinitis, Lemierre syndrome, necrotizing cervical fasciitis, carotid artery aneurysm, sepsis and even death (1). The estimated mortality rate due to DNIs is 0.3-1.6% (2). Deep neck abscesses occur in the potential spaces between the cervical fasciae, and the complex anatomy of the neck can obscure the severity of the disease (1,3). In the pre-biotic era, 70% of DNIs were caused by tonsillitis, and DNIs were seen much more frequently (4). However, in the recent literature, there has been a decline in tonsillitis-related DNIs and a relative increase in odontogenic causes in the adult population (1).

In this case series, three patients with advanced odontogenic DNIs are discussed in light of the current literature.

## CASE PRESENTATIONS

Written informed consent for case reports was obtained from each of three patients.

### CASE 1

**Figure 1:**
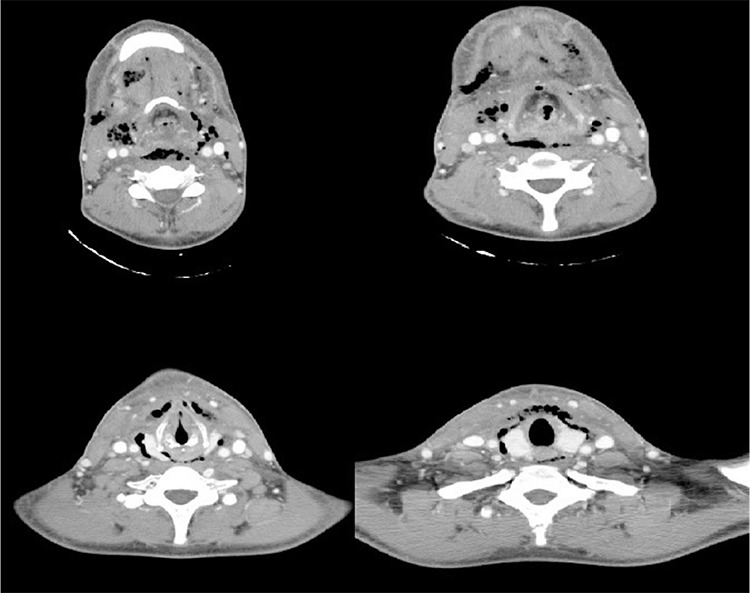
Serial computerized tomography scans of the first patient showing multi-space abscess formation with air bubbles.

**Figure 2:**
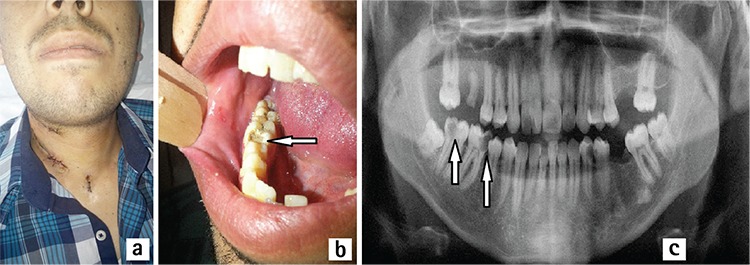
Clinical appearance of the first patient after decanulization from tracheotomy was shown (a). Arrow shows the intraoral lytic lesion in first molar tooth which can be the possible source of deep neck infection (b). Orthopantogram of the first patient was demonstrated (arrows show the lytic lesions in the first and second molar teeth) (c).

A twenty-three year old otherwise healthy male patient was admitted to the emergency department with swelling in the neck and pain in the right submandibular region. He had a history of oral using of amoxicillin-clavulanic acid (Augmentin^®^, Glaxo Smith Kline drugs; USA) treatment for four days for right mandibular first molar tooth pain. His general status had gone worse in the last 24 hours with increased fever and trismus. He had no respiratory distress. In his physical examination; he had 38.5 °C fever, trismus preventing oral examination and moderate aryepiglottic fold edema on fiber optic nasal endoscopic examination. Neck stiffness and pain during palpation was also present. He had 21x10^3^/µL. white blood count. Neck computerized tomography (CT) was performed. There was a fluid collection with disseminated gas bubbles in all compartments of the neck also including prevertebral space; right side was affected more than the left side (Figure 1). Since he refused the risk of tracheotomy during operation, right submandibular region abscess was drained under local anesthesia. Intravenous ampicillin-sulbactam 4x1.5 gr/day (Ampisid^®^, Mustafa Nevzat Drugs; Turkey) with combination of clindamycin (Klindan^®^, Bilim Drugs; Turkey) 3x600 mg/day was started. The following day; he had respiratory distress. His fiber optic nasal endoscopic examination revealed prominent epiglottic edema. In his control neck CT, there was fluid collection in anterior cervical visceral layer and right carotid space with air bubbles without mediastinal involvement. Tracheotomy was performed under local anesthesia, purulent material in the anterior visceral space drained spontaneously during surgery. Right carotid space abscess was also drained with blunt dissection with a second incision 2 cm. upper from tracheotomy incision. After this second surgery, his clinical situation got better. The next day his white blood cell count decreased to 11x10^3^/µL. His body temperature was in normal limits. *Streptococcus viridans* which was sensitive to clindamycin was isolated from the culture. Five days later laryngeal edema and trismus recovered totally and decanulization from tracheotomy was performed (Figure 2a). The patient was discharged from the hospital after 14 days of intravenous treatment with ampicillin-sulbactam and clindamycin combination. After trismus has resolved, lytic lesions in right mandibular first and second molar teeth which were the probable causes of infection were seen (Figure 2b, 2c). He was sent to a dentist for the treatment of first and second molar teeth. Three months of follow-up was uneventful.

### CASE 2

**Figure 3:**
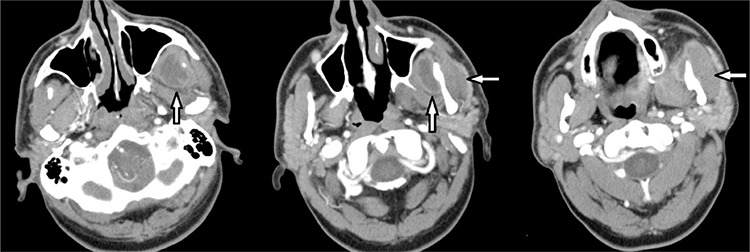
Computerized tomography scans of the second patient were presented (vertical arrows show the medial pterygoid, horizontal arrows show the parotid region abscess formation).

**Figure 4:**
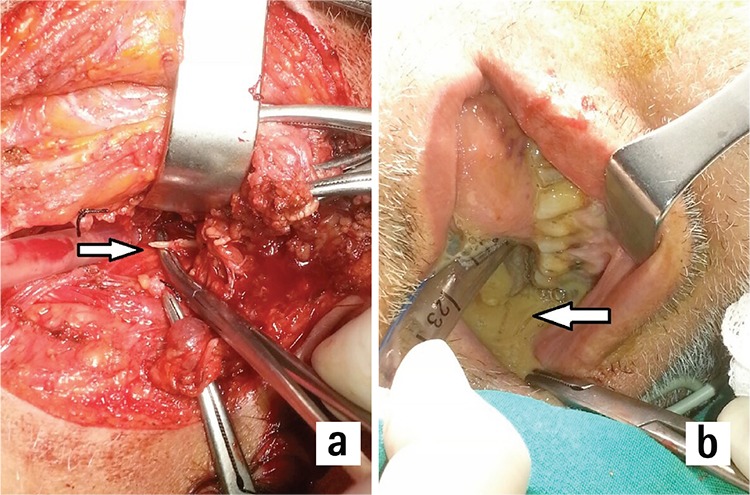
Parapharyngeal space of the second case after extraction of the submandibular gland was shown (arrow shows the styloid process) (a). Arrow shows the spontaneous rupture of the abscess into the oral cavity after blunt dissection in the parapharyngeal space (b).

A fifty-two-year old type II diabetic patient was admitted to the emergency department with pain and swelling in the left pre-auricular and facial region. He had previous treatment with oral 500 mg cefuroxime axetil (Aksef^®^, Nobel Drugs; Turkey) for five days because of a left mandibular second molar tooth infection. On his physical examination there was a swelling in the left parotid region, he had also trismus preventing oral examination. He had no respiratory distress. With fiber optic nasopharyngoscopic examination, he had no laryngeal edema. Blood count revealed 17x10^3^/µL. white blood cell count with blood glucose level of 325 mg/dL. Neck CT of the patient revealed parotid region abscess with involvement of medial pterygoid muscle and parapharyngeal space on the left site (Figure 3). After fiber optic intubation under general anesthesia, superficial parotidectomy and parotid abscess drainage was performed. For medial pterygoid abscess, with extraction of left submandibular gland, styloid process was seen and parapharyngeal space was achieved (Figure 4a). After blunt dissection, the medial pterygoid abscess spontaneously ruptured into the oral cavity (Figure 4a). There was no facial paralysis or paresis in the postoperative period. Close monitoring of the blood glucose level was done by subcutaneous insulin treatment in the postoperative period after Endocrinology department consultation. For antibiotic treatment, intravenous ampicillin-sulbactam 4x1.5 gr/day (Ampisid^®^, Mustafa Nevzat Drugs; Turkey) with combination of gentamycin (Genta^®^, İbrahim Etem Ulagay Drugs; Turkey) 2x80 mg/day was administered. *Streptococcus viridans* and *Klebsiella pneumonia* which was sensitive to gentamycin was isolated from the cultures. After 10 days of intravenous treatment he was discharged and sent to a dentist for treatment of left second mandibular molar tooth decay. He had no more complaints after six months of follow-up.

### CASE 3

**Figure 5:**
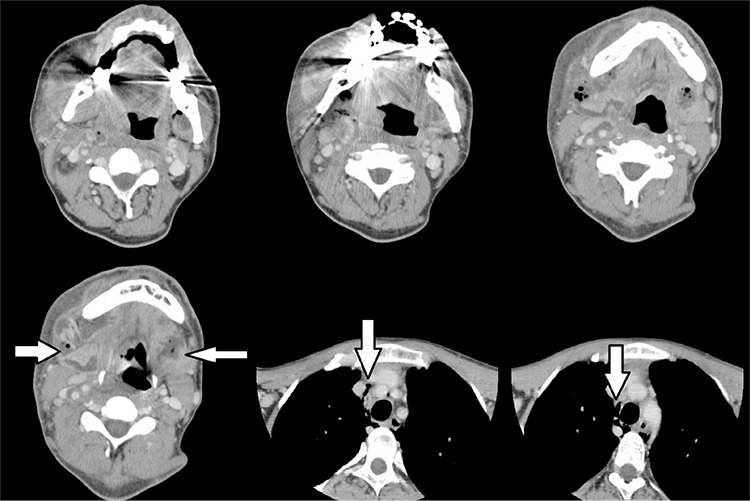
Serial computerized tomography scans of the neck and thoracic region for the third case has been demonstrated (horizontal arrows show the bilateral submandibular abscess formations, vertical arrows show the air bubbles in the upper mediastinum).

**Figure 6:**
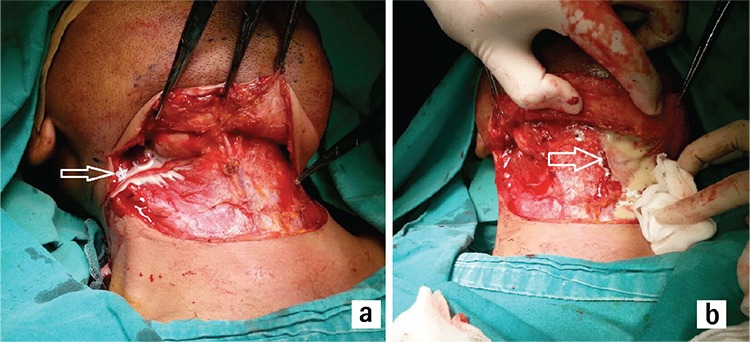
Arrow shows drainage of the right submandibular abscess (a). Arrow shows drainage of the left submandibular abscess (b).

**Figure 7:**
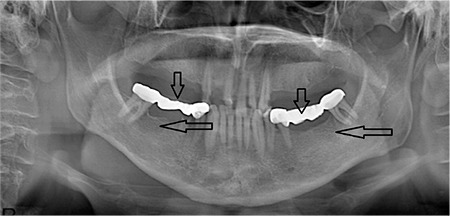
Orthopantogram of the third patient was presented (vertical arrows show the silver amalgam restorations, horizontal arrows show the radiolucent periapical infections in the root of third molar teeth).

Thirty-two years old male patient admitted to the emergency department with a swelling in the neck and pain in bilateral mandibular third molar teeth which were packed with amalgam dental fillings before. In his medical history; he had anticonvulsant treatment for epilepsy and he had mild mental retardation. He had used oral amoxicillin-clavulanic acid (Augmentin^®^, Glaxo Smith Kline drugs; USA) for three days and intramuscular sulbactam-ampicillin 1 gr. (Ampisid^®^, Mustafa Nevzat Drugs; Turkey) twice a day for three days for bilateral mandibular third molar teeth pain. He had 38.3 0C fever without respiratory distress, trismus, dyspnea or thoracic pain. White blood cell count was 14.3x103/µL. There was 3x3 cm. painful swelling hard on palpation in both submandibular region. Indirect laryngoscopic examination revealed moderate laryngeal edema mostly on right aryepiglottic fold. On his neck and thoracic CT, there was collection mainly in both submandibular regions, also involving multiple compartments of the neck with air bubbles. Air bubbles were descending to the level of upper mediastinum with increased density of mediastinal fat (Figure 5). Bilateral cervicotomy with Kocher’s incision was performed under general anesthesia and bilateral submandibular abscess was drained (Figure 6). With blunt dissection through anterior cervical region and anterior visceral fascia of trachea, upper mediastinum was also reached. There was no fluid collection in this region. Immediately following surgery, tracheotomy with separate incision was performed due to respiratory failure. Although there was no isolated microorganism in the culture, ertapenem 1 gr. (Invanz^®^, Merck sharp and Dohme Drugs; USA) once a day was started by Infectious disease department for mediastinitis complication of a ODNI. In the postoperative period, the patient got well with normal body temperature, and white blood cell count decreased to 9x10^3^/µL. in the postoperative fifth day. On his control neck and thoracic CT in the postoperative seventh day, cervical abscess and mediastinitis was totally recovered. There was only small number of air bubbles in neck compartments. His orthopantogram is shown in Figure 7. Decanulization of tracheotomy was performed on eleventh day and the patient was discharged on 14^th^ day. He was sent to a dentist for treatment of mandibular teeth. He retained well on the first month control.

## DISCUSSION

Although the primary cause of DNI was tonsillopharyngeal infection (70-80% of all cases) before the widespread use of antibiotics, in time, the prevalent source of DNIs has been confirmed to be odontogenic in both non-elderly and elderly adult patients (5). In a recent broad series (6), 80% of DNI cases were caused by dental infections. Odontogenic DNIs (ODNIs) are medical and surgical challenges for clinicians with regard to the optimal treatment plan and they are mostly underestimated (1,4,5). The molar teeth are the most common source of ODNIs, and the mandibular molar teeth are affected more commonly than the maxillary molar teeth (4). The second and third mandibular molar teeth are important sources of DNIs, because their roots extend to the junction of the mylohyoid muscle with the mandibular corpus adjacent to the submandibular and parapharyngeal spaces, making the submandibular space the most commonly involved area in DNIs (5,7). In our patients, case 1 had first molar, case 2 had second molar, and case 3 had third molar tooth infection. The predisposing factors for ODNIs include bad oral hygiene, dental calculus, plaques and inappropriate inlays (4,5). All of our patients had bad oral hygiene and case 3 had amalgam dental filling, which may be a source of infection. Although diabetes mellitus is thought to be a predisposing factor for morbidity and mortality in ODNIs (8), mortality can be seen in young patients without additional diseases (2). Pregnancy may be another important factor, which can impair the immune response of the patient causing a dramatic increase in abscess formation and fetal mortality (7).

Urgent and thorough evaluation of the ODNI is mandatory to avoid life-threatening complications such as mediastinitis, sepsis and carotid artery rupture (1). Imaging studies should be performed when deep neck abscess is suspected. Ultrasound is cheap and easily available, but it is subjective and cannot show a deep or small abscess. Magnetic resonance imaging is expensive, needs more time during scanning than CT, and has similar prognostic values to CT. Contrast-enhanced tomography can differentiate a cellulitis from an abscess and it provides a good anatomical relationship for surgery. An encapsulated hypodense heterogeneous lesion with more hypodense central area may indicate an abscess formation (9). Although CT seems to be the gold standard imaging technique for evaluation of DNIs, its positive correlation with surgical findings is between 68-83% (9). Recently, Freling et al. (10) found the positive predictive value for CT findings in deep neck abscesses as 82%, and they found that air bubbles with or without fluid collection indicated a higher percentage of abscess formation. In our patients, the CT findings of air bubbles in case 1 and 3 indicated abscess formation.

Mediastinitis is a rare, life-threatening complication of DNIs. In most of cases, the primary etiology is ODNIs and the mortality rate is 25-40% (11). The absence of barriers between the cervical fasciae and the mediastinum facilitates extension of the infection to thoracic inlet. There are three potential pathways for spread of a neck infection into the mediastinum: 1) The pretracheal route to the anterior mediastinum, 2) The lateral pharyngeal route to the middle mediastinum, and 3) The retropharyngeal-retro-visceral route to the posterior mediastinum (12). The pretracheal route was the main route for spread of infection to mediastinum in our case 3. Thoracic pain, jugular distention, dyspnea, hypoxia and respiratory failure are the cardinal findings of mediastinitis, but the clinical signs and symptoms may be subtle. In the series of Mora et al., (11) seven of 15 patients had no dyspnea or hypoxia. They emphasized that mediastinitis should not be ruled out when there is absence of respiratory distress. In the same manner, in the series by Boscolo-Rizzo et al. (8), the diagnosis in 10 of 16 patients with mediastinitis due to DNIs was made based on CT findings without clinical signs of respiratory stress. Our case with mediastinitis did not have dyspnea or hypoxia either. Evaluation of CT findings is also important for the diagnosis of mediastinitis, as in ODNIs. The primary CT findings are fluid collection and free gas bubbles in the mediastinum for the diagnosis. Some of the secondary findings include increased attenuation of mediastinal fat, enlarged mediastinal lymph nodes and pleural effusions (13). In the series of Exarhos et al. (13), all patients with mediastinitis had mediastinal fat attenuation and free gas bubbles were seen in 57.5% of the cases. The CT findings of our case 3 also had free gas bubbles in the upper mediastinum and mediastinal fat attenuation. The timing and type of approach of thoracic surgery in the case of mediastinitis complicating an ODNI are controversial. Some authors (14) strictly recommend early aggressive thoracic surgery regardless of the level of infection, whereas others (11,12) recommend mostly a trans-cervical approach; they recommend thoracic surgery when the infection spreads in a cranio-caudal level lower than the upper mediastinum or when more than one space in the thorax are involved. In our case 3, only the trans-cervical approach was undertaken because only the upper mediastinum was affected, and there was no fluid during blunt dissection in the upper mediastinum. Notably, the clinical and radiological follow-up of a patient with ODNI is important when choosing candidates for surgical exploration. When surgical exploration is the treatment of choice, anesthesia is very critical. Swelling of the soft tissues in the neck and oropharyngeal edema can limit the extension of the head and make the tracheal intubation under general anesthesia difficult. Furthermore, cases of pharyngeal wall abscess rupture to the oral cavity during general anesthesia causing aspiration have been presented (15). In case 2, spontaneous abscess rupture to the oral cavity was also observed. Tracheotomy under local anesthesia is the gold standard airway management for DNIs (1). However, it can also be difficult due to anatomical distortion of the anterior neck compartments (15), such as abscess formation in the anterior cervical plains, as in our case 1. Ovassapian et al. (15) recommend fiber optic intubation under topical anesthesia for managing the airway in patients with DNIs in order to prevent the complications of tracheotomy and airway collapse under general anesthesia. In our case 2, we also managed the airway with fiber optic intubation, but we preferred general anesthesia. When tracheotomy is the choice for managing the airway, an incision different to the cervical incision should be made for preventing the descending of infection to the mediastinum (1). We also performed tracheotomy with separate incisions for cases 1 and 3. When general anesthesia and tracheal intubation can be achieved successfully, the patient can still have the risk of airway compromise immediately after surgery in cases with descending mediastinitis. In the series of Mora et al. (11), six of 21 patients with descending mediastinitis needed tracheotomy for airway management early after the surgical intervention. We performed tracheotomy due to airway collapse in the early postoperative period in our case 3 who also had mediastinitis.

For DNI cases, culture-directed antibiotic treatment is the mainstay of treatment, in addition to surgical drainage. Before the results of the cultures are obtained, empirical antibiotic treatment must be begun to prevent progression of the infection (16). The most commonly isolated microorganisms are the alpha hemolytic streptococcus group, in particular, *Streptococcus viridans* (8,16). This bacterium was also isolated in our cases 1 and 2. However, recently, in a series of 634 patients by Celakovsky et al. (6), Streptococcus pyogenes was the most commonly isolated bacterium. *Staphylococcus, Klebsiella pneumonia*, anaerobic *Bacteroides* and *Peptostreptoccocus* were the other common bacteria isolated from cultures of DNIs (1,16). Anaerobic coverage is required to a higher extent in ODNIs than in tonsillopharyngitis-related DNIs. In the series of Huang et al. (16), the rate of anaerobic *Peptostreptoccocus* isolation was significantly higher in DNIs of dental origin than in upper airway infection related DNIs. For optimal empirical coverage, either penicillin with a β-lactamase inhibitor such as amoxicillin or ticarcillin with clavulanic acid, or a β-lactamase-resistant antibiotic such as cefoxitin, cefuroxime, imipenem or meropenem in combination with an anaerobic-effective antibiotic (such as clindamycin or metronidazole) are recommended (1). We preferred the combination of ampicillin-sulbactam and clindamycin in case 1. Diabetes mellitus is an important predictor of complications of DNIs, it is a significant risk factor for infection-related morbidity and mortality. The hyperglycemic state can cause leukocyte, macrophage and fibroblast impairment, which render the patient prone to serious infections. Therefore, meticulous control of the hyperglycemic state is crucial in DNI management, and the use of insulin rather than oral hypoglycemic drugs is the best option for controlling the blood glucose levels in these patients (8). In diabetic patients, the isolated microorganisms from the pus samples are conflicting. Peripheral vascular disease in diabetics may predispose patients to anaerobic infections (8). On the other hand, Celakovsky et al. (6) found that the incidence of anaerobic bacteria was higher in non-diabetic patients. *Klebsiella pneumonia* was the leading cause of DNIs in diabetic patients in some series (1,16); it was also the most commonly isolated organism in the series of Wang et al. (17) regardless of the diabetes mellitus. The importance of *Klebsiella pneumonia* is its resistance to clindamycin. When *Klebsiella pneumonia* is suspected, gentamycin should be added to the empirical treatment protocol (1,17). In our diabetic case 2, this bacteria was also isolated and successfully treated with gentamycin 80 mg twice a day in addition to sulbactam-ampicillin 1.5 gr four times a day and subcutaneous insulin treatment. Despite improvements in the culture techniques, bacteria isolation may not be successful in 10% of the patients. The major cause of this situation is previous use of antibiotics, especially through the intravenous route before surgical drainage (16). In cases with ODNI-related mediastinitis, carbapenem is effective for both aerobes and anaerobes for the initial empirical treatment (12). In case 3, he also had a history of previous oral and intramuscular antibiotic use, which can be the leading cause of false-negative culture results. We chose ertapenem, a parenteral group 1 carbapenem, for the empirical treatment of ODNI- related mediastinitis in case 3.

In conclusion, an underestimated tooth infection can cause hazardous complications of ODNIs such as airway compromise and mediastinitis. Prompt and thorough evaluation should be performed in order to prevent these complications. To emphasize the important issues in our cases:

- Contrast-enhanced CT is the imaging technique of choice for determining abscess formation and the extent of infection.

- Symptoms can be subtle in cases with mediastinitis, and false-negative cultures can be encountered, especially in patients with a history of previous antibiotic use.

- When the infection is limited in the upper mediastinum in ODNI cases, a trans-cervical approach is sufficient for controlling the mediastinitis.

- Tracheotomy is the gold standard for the airway management of cases with DNIs; however, spontaneous abscess rupture can be observed when there is abscess formation in the anterior cervical visceral layer. Fiber optic intubation can be attempted in suitable cases to prevent complications of tracheotomy.

- In diabetic patients, *Klebsiella pneumonia*-directed gentamycin may be added to the empirical treatment regimen. Controlling the blood glucose levels in these patients via insulin administration is important to prevent the spread of infection. Ertapenem is a good choice for the empirical treatment of ODNI-related mediastinitis cases.
